# Quantitative and Qualitative Assessment of Urinary Activity of ^18^F-Flotufolastat-PET/CT in Patients with Prostate Cancer: a Post Hoc Analysis of the LIGHTHOUSE and SPOTLIGHT Studies

**DOI:** 10.1007/s11307-023-01867-w

**Published:** 2023-11-06

**Authors:** Phillip H. Kuo, Rick Hermsen, Ross Penny, Ernst J. Postema

**Affiliations:** 1 Departments of Medical Imaging, Medicine, and Biomedical Engineering, University of Arizona, Tucson, AZ USA; 2 Southern Arizona Veterans Administration Healthcare System, Tucson, USA; 3 Department of Nuclear Medicine, Canisius Wilhelmina Hospital, Nijmegen, The Netherlands; 4 Blue Earth Diagnostics Ltd, Oxford, UK

**Keywords:** Prostatic neoplasms, Positron emission tomography, PSMA, rhPSMA, Urinary activity, Bladder, Halo artifact

## Abstract

**Purpose:**

To evaluate the impact of urinary activity on interpretation of ^18^F-flotufolastat (^18^F-rhPSMA-7.3) PET/CT, we conducted a post hoc qualitative and quantitative analysis of scans acquired in two phase 3 studies of ^18^F-flotufolastat.

**Procedures:**

Newly diagnosed or recurrent prostate cancer patients enrolled in LIGHTHOUSE (NCT04186819) or SPOTLIGHT (NCT04186845), respectively, underwent PET/CT 50–70 min after intravenous administration of 296 MBq ^18^F-flotufolastat. For the present analysis, 718 ^18^F-flotufolastat scans (352 from LIGHTHOUSE and 366 from SPOTLIGHT) were re-evaluated by three board-certified nuclear medicine physicians. Reader 1 performed a quantitative assessment (*SUV*_*max*_ and *SUV*_*mean*_) of bladder activity in a circular region-of-interest over the maximum diameter of bladder activity in the transverse plane. All three readers qualitatively assessed the impact of any urinary activity in the bladder on image interpretation using a three-point scale (0 = no/minimal visible urinary activity, 1 = urinary activity visible but distinction between urine and disease possible and 2 = assessment inhibited by urinary activity) and the presence/absence of ureteric activity and halo artifacts.

**Results:**

In total, 712/718 scans were evaluable. Reasons for exclusion were cystectomy, renal failure, or urinary catheter in situ (*n* = 2 each). The median bladder *SUV*_*max*_ and *SUV*_*mean*_ were 17.1 and 12.5, respectively. By majority read, 682/712 (96%) patients had either no urinary activity (score = 0) or visible activity that could be distinguished from disease uptake (score = 1). In the minority of patients (24, 3.4%) where urinary activity did impact assessment (score = 2), the median bladder *SUV*_*mean*_ was higher (20.5) than those scored 0 (3.8) or 1 (14.0). Ureteric activity was absent in 401 (56%) patients. Halo artifacts were observed in only two (0.3%) patients (majority read).

**Conclusions:**

^18^F-Flotufolastat urinary activity did not influence disease assessment for the majority of patients. While this study was not designed as a head-to-head comparison, the median bladder SUVs are lower than previously reported values for other renally cleared PSMA-PET radiopharmaceuticals.

**Supplementary Information:**

The online version contains supplementary material available at 10.1007/s11307-023-01867-w.

## Introduction

Prostate-specific membrane antigen (PSMA) positron emission tomography (PET) has become a mainstay for diagnostic imaging of primary and recurrent prostate cancer [1, 2].

Novel ^18^F-labeled radiohybrid (rh) PET radiopharmaceutical, ^18^F-rhPSMA-7.3 (^18^F-flotufolastat), is a newly FDA-approved high-affinity PSMA-targeting diagnostic for patients with prostate cancer. The diagnostic performance and safety of ^18^F-flotufolastat have recently been investigated in two phase 3 clinical trials. The LIGHTHOUSE study (NCT04186819) investigated ^18^F-flotufolastat in men newly diagnosed with prostate cancer who were scheduled for radical prostatectomy with pelvic lymph node (LN) dissection [3], and the SPOTLIGHT study (NCT04186845) evaluated ^18^F-flotufolastat in men with biochemical recurrence following curative intent treatment of localized prostate adenocarcinoma [4].

The normal biodistribution of PSMA radiopharmaceuticals can influence the interpretation of PET images, particularly in the prostate/bed and pelvic LN regions where excreted urine can visually conceal the anatomical areas under assessment or appear visually indistinguishable from disease [5–7]. Approaches to minimize the impact of bladder activity on image interpretation, such as early/late imaging and/or co-administration of diuretics, have been investigated with varying results [8–12]. However, to ensure patient comfort and optimal integration into an existing workflow, ideally, a scanning protocol would require neither approach.

Early clinical data with ^18^F-flotufolastat showed it to have lower average urinary excretion than reported values for other renally cleared PSMA-PET radiopharmaceuticals [13]. Therefore, a potential exists for improved image evaluation in the prostate and peri-ureteric regions. Indeed, retrospective data from routine clinical use of ^18^F-flotufolastat at institutions in Germany show there to be good distinction between the primary tumor and background bladder activity in patients with primary prostate cancer undergoing N-staging [14].

Here, we report the findings of a post hoc analysis of the LIGHTHOUSE and SPOTLIGHT study scans to quantitatively and qualitatively assess the urinary activity in bladder and ureters in patients undergoing ^18^F-flotufolastat-PET to assess potential for impact on disease assessment.

## Materials and Methods

### Patient Population

The LIGHTHOUSE and SPOTLIGHT studies were performed in line with the principles of the Declaration of Helsinki. Approval was granted by the Ethics Committees of the participating institutions, and all patients provided written informed consent. No further ethical approval was required for this post hoc analysis.

All evaluable patients from the efficacy populations of the recently conducted phase 3 LIGHTHOUSE (NCT04186819) and SPOTLIGHT (NCT04186845) studies were included in the analysis. A total of 718 PET scans were analyzed. This total comprised scans from all 352 patients with treatment-naïve, newly diagnosed, unfavorable intermediate to very high–risk prostate cancer who had an evaluable ^18^F-flotufolastat-PET as part of the LIGHTHOUSE study in addition to all 366 evaluable patients with biochemical recurrence of prostate cancer included in the SPOTLIGHT study.

### Radiopharmaceutical

The patients enrolled in LIGHTHOUSE were administered a median (range) activity of 307.3 MBq (213.5–397.8 MBq) ^18^F-flotufolastat, while patients in SPOTLIGHT received 306.0 MBq (230.1–355.2 MBq), as previously reported [3, 4]. Co-administration of diuretics was not permitted in either study. Patients had been asked to be well hydrated prior to the administration, and scanning departments were not prevented from following any local hydration practices during the uptake period; no guidance was provided on any volumes of hydration, and patients were encouraged to void immediately prior to entering the scanning room. No additional radiopharmaceutical administration was required for the present analysis.

### PET/CT

All patients in LIGHTHOUSE and SPOTLIGHT underwent PET/computed tomography (CT) 50–70 min after intravenous administration of ^18^F-flotufolastat [3, 4]. Acquisition and reconstruction parameters were typical for fluorinated radiopharmaceuticals and had been independently approved by the studies’ imaging core lab [3, 4]. Iterative and time-of-flight reconstructions were permitted, but more advanced algorithms, such as point-spread function recovery, were not.

### Image Analysis

For the present post hoc analysis, a further three readers evaluated the LIGHTHOUSE and SPOTLIGHT scans in order to determine the potential of any urinary activity to interfere with assessment. For instance, the readers assessed if radioactivity in the ureter might be mistaken for a lymph node metastasis or if assessment at the vesico-urethral junction, urethra, or prostate/bed may be inhibited by highly concentrated urinary activity. Reader 1 was a nuclear medicine physician with 25 years’ experience, Reader 2 was a nuclear medicine physician with 13 years’ experience, and Reader 3 was a dual-certified nuclear medicine physician/radiologist with 19 years’ experience. All readers had completed ^18^F-flotufolastat reader interpretation training as part of the original studies, which was supplemented with specific methodology training for this post hoc analysis. The training did not include any cases from the study population. Reader 1 performed both quantitative and qualitative assessments of each scan, while readers 2 and 3 performed qualitative assessments only. All assessments were undertaken in isolation. For this post hoc read, the readers were blinded to all clinical data including the results of any additional imaging the patient received. Image analysis was undertaken on MIM version 7.1.8 (MIM Software Inc., Cleveland, OH), with the CT provided for anatomical reference only.

### Quantitative Analysis

To quantitatively assess activity in the bladder, reader 1 placed a circular region of interest (ROI) over the maximum radioactive diameter in the bladder in the transverse plane and recorded the *SUV*_*max*_ and *SUV*_*mean*_. This was done in such a way that the ROI was as large as possible but still contained within the radioactive extent of the bladder (Supplemental Fig. 1).

### Qualitative Analyses

**Fig. 1:**
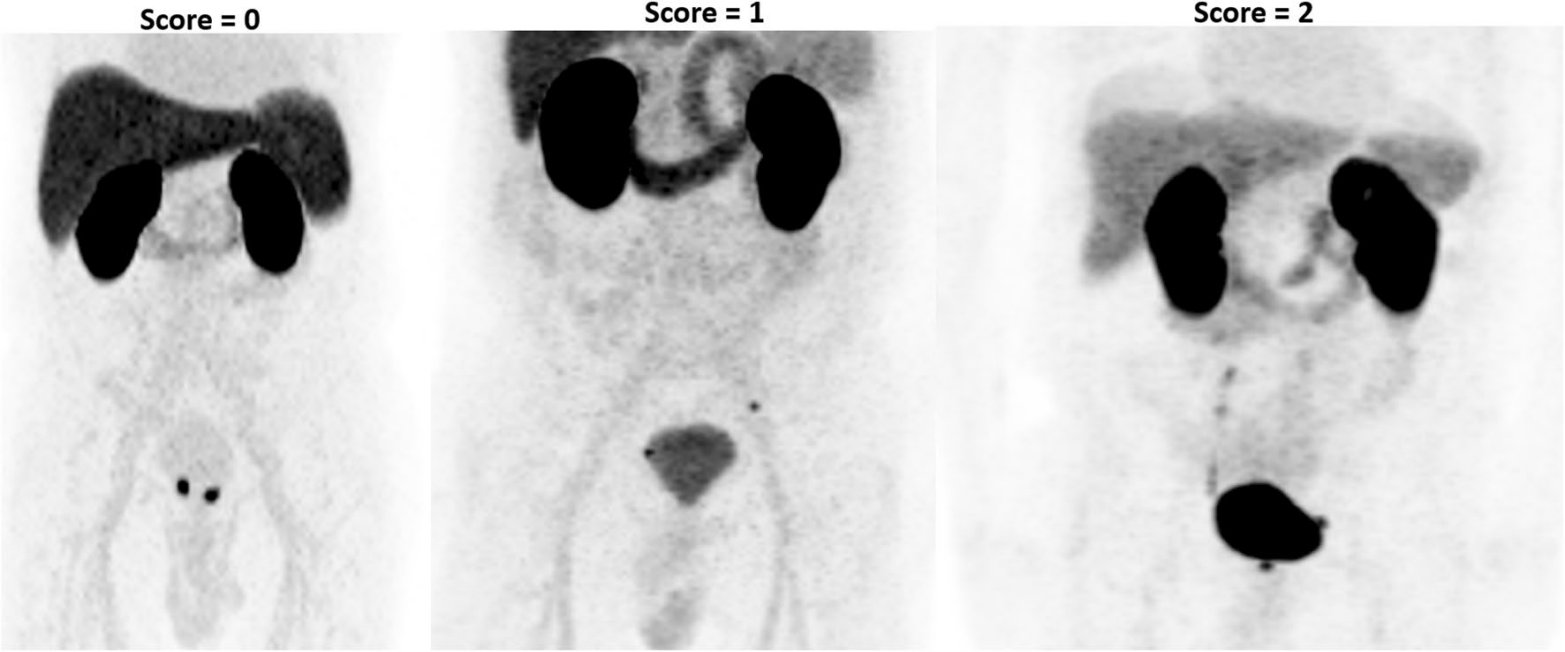
Representative scoring in the qualitative bladder activity analysis. Maximum intensity projection images captured at SUV 0–10

All three readers independently conducted a qualitative assessment of the extent to which urinary activity in the bladder affected assessment of prostate/prostate bed and para-iliac LNs. The full read methodology is provided in Supplemental Table 1. Qualitative assessments were made using a three-point scale (detailed below and with example images in Fig. 1) and with reference to the maximum intensity projection (MIP) and three planar reconstructions — low-dose CT was used for anatomical reference only.

0. No or hardly any urinary activity visible: light grey activity in the bladder when the scan is read at a SUV scale of 0–10.
1. Urinary activity visible, but distinction between urine and disease possible:
  Linear activity in the ureters and separately visible nodular-shaped uptake in keeping with LNs or no LNs
  Activity in the bladder, but primary or local recurrence or LNs close to bladder clearly separate from bladder
  Activity in the urethra, easily distinguishable from primary or local recurrence in the prostate bed
2. Assessment inhibited by urinary activity:
  Reader not able to tell (without consulting other modalities) if a single focus of activity is either a LN or stasis of urine in the ureter
  Reader unable to separate bladder from disease

#### Ureters

**Fig. 2:**
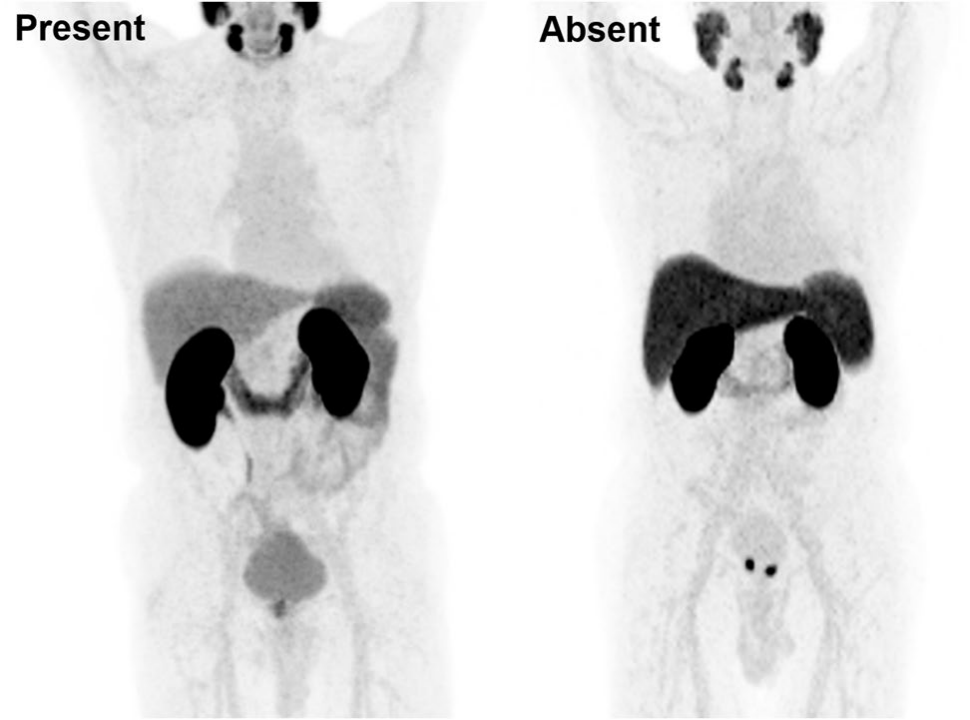
Representative qualitative assessment of the presence and absence of ureteric activity. Maximum intensity projection images captured at SUV 0–10

In order to explore the presence of urinary activity in the ureters, the three readers independently assessed the MIP at a SUV scale of 0–10 for the *presence* or *absence* of a stasis of urine in keeping with ureteric activity regardless of the presence or absence of pathological nodular activity (i.e., LNs). Example images are provided in Fig. 2.

#### Halo Artifacts

**Fig. 3:**
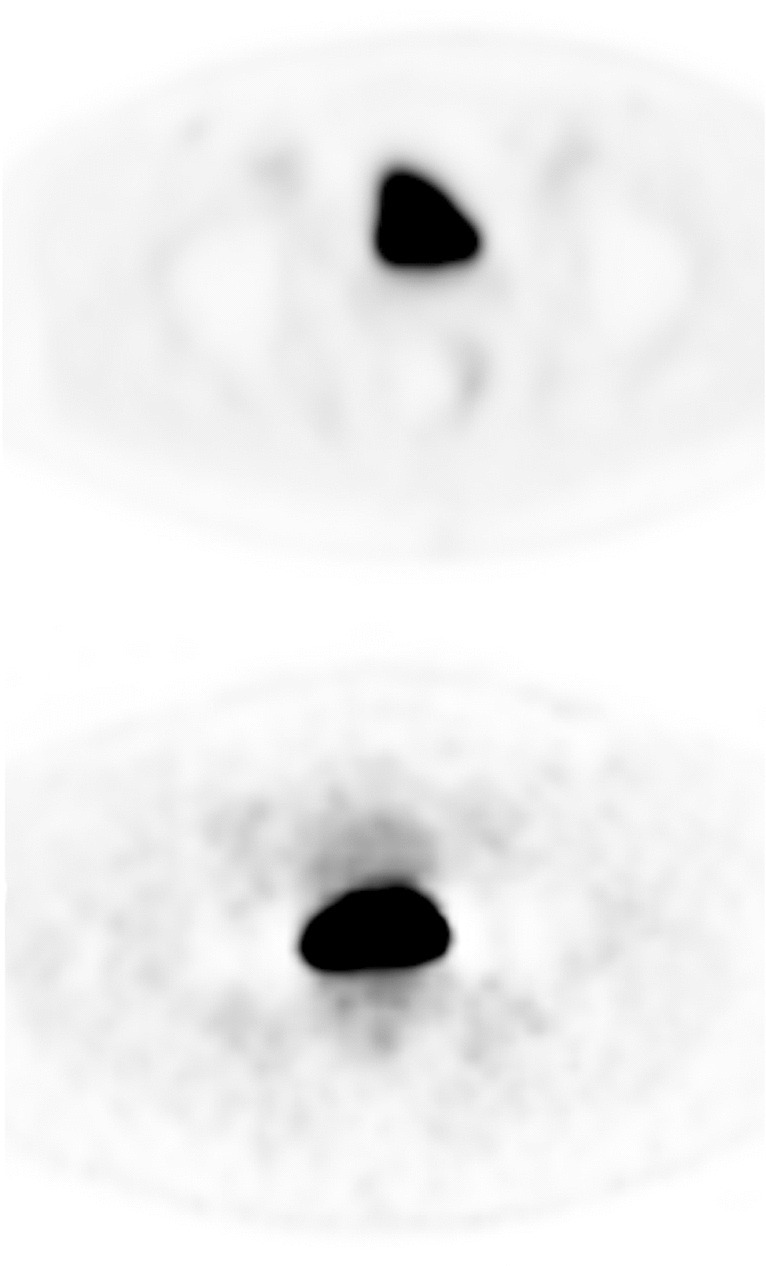
Qualitative assessment of the presence of halo artifacts. The figure presents ^18^F-flotufolastat-PET images from two patients (top and bottom, respectively) in which the majority (2/3) of readers agreed on the presence of a halo artifact (SUV scale 0–10). On the first image, the halo artifact is seen around the entire bladder, whereas in the second image, the halo is only seen laterally from the bladder

In order to explore the presence of halo artifacts around the bladder, the three readers independently assessed transverse slices for the *presence* (Fig. 3) *or absence* of a gross photopenic region extending significantly beyond the bladder and overlaying the other structures of the pelvis.

### Statistics

All results were summarized as a percentage for each reader and as majority read, defined as agreement between at least any two out of three readers.

## Results

Of the 718 patients included in this study, 712 (99%) were evaluable for bladder activity. Six patients could not be evaluated because of a history of cystectomy (*n* = 2); renal failure (*n* = 2); and urinary catheter in situ (*n* = 2). Of these 712 evaluable patients, 348 had newly diagnosed prostate cancer and 364 had biochemical recurrence.

### Quantitative Assessment of Bladder Activity

**Fig. 4:**
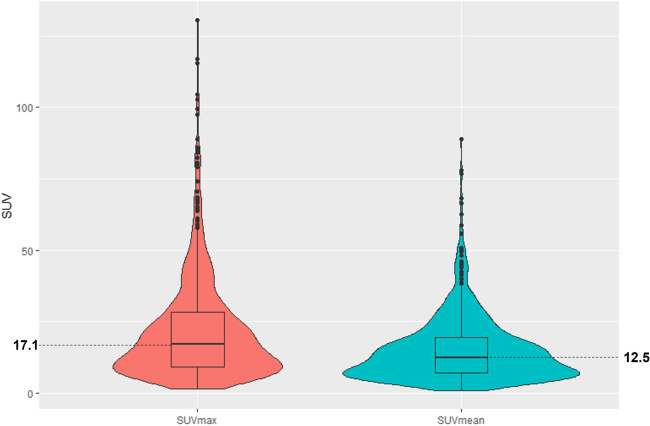
Quantitative assessment (*SUV*_*max*_ and *SUV*_*mean*_) of bladder activity

As shown in Fig. 4, the median *SUV*_*max*_ in the bladder was 17.1 (range, 1.3–130.6; interquartile range [IQR] 9.2–28.3). The median *SUV*_*mean*_ was 12.5 (range, 0.7–88.7; IQR, 7.0–19.3).

### Qualitative Assessment of Bladder Activity

Figure 1 presents representative images that were rated 0, 1, or 2 by all readers. Across the three readers, between 616 and 694 of the 712 patients (87–97%) were scored 0 or 1 as the reader determined it was possible to distinguish between urinary activity and disease (Table 1). By majority read, 96% (682/712) of scans were scored 0 or 1. Of the 77 scans scored 0 by majority read, 47 were scored 0 by 3/3 readers, and 30 by 2/3 readers. Of the 605 scans scored 1 by majority read, 464 were scored 1 by 3/3 readers and 141 by 2/3 readers. For a small minority of patients (18–96/712 [3–13%]), the assessment was inhibited by urinary activity (score = 2), the majority read being 3.4% (24/712), of which two patients were scored 2 by 3/3 readers and 22 by 2/3 readers. Six scans (0.8%) were tied (*i.e.*, all three readers assigned a different score).

**Table 1:** Qualitative assessment of bladder activity by reader and for the majority read with SUV stratification

Qualitative score	0	1	2	Tie
Reader 1				
* N* (%)	84 (12%)	532 (75%)	96 (13%)	NA
Reader 2				
* N* (%)	94 (13%)	600 (84%)	18 (3%)	NA
Reader 3				
* N* (%)	59 (8%)	600 (84%)	53 (7%)	NA
Majority read				
* N* (%) Median bladder *SUV*_*mean*_^a^(IQR) Median bladder *SUV*_*max*_^a^(IQR)	77 (11%) 3.8 (2.9–4.5) 5.2 (4.1–5.9)	605 (85%) 14.0 (8.4–20.3) 18.8 (11.3–29.9)	24 (3.4%) 20.5 (12.4–28.0) 25.6 (16.5–40.1)	6 (1%) 5.4 (4.9–5.9) 7.0 (6.8–7.6)

0 = no visible urinary activity, 1 = urinary activity visible, but distinction between urine and disease possible and 2 = assessment inhibited by urinary activity. Tie = all three readers assigned a different score

*NA* not applicable, *IQR* interquartile range

^a^Using SUV data from Reader 1’s analysis

Using the SUV data captured by reader 1, the median *SUV*_*max*_ and *SUV*_*mean*_ were calculated for the majority read qualitative scores (Table 1). These data show the median bladder *SUV*_*mean*_ to be higher for those scored 2 compared with those scored 0 or 1.

### Qualitative Assessment of Ureteric Activity

Stasis of urine was absent in 56% (401/712) of cases (majority read) as shown in Table 2. Figure 2 shows examples of cases scored present or absent for ureteric activity.

**Table 2:** Qualitative assessment of ureteric activity by reader and for the majority read

Qualitative score	Yes	No
Reader 1		
* N* (%)	253 (36%)	459 (64%)
Reader 2		
* N* (%)	309 (43%)	403 (57%)
Reader 3		
* N* (%)	369 (52%)	343 (48%)
Majority read		
* N* (%)	311 (44%)	401 (56%)

### Qualitative Assessment of Halo Artifacts

The majority read confirmed the presence of a halo artifact around the bladder in only two cases (0.3%). The reads for all individual readers are summarized in Table 3. Figure 3 shows the two cases in which the majority (2/3 readers) agreed on the presence of a halo artifact.

**Table 3:** Qualitative assessment of the presence of halo artifacts by reader and for the majority read with SUV stratification

	Yes	No
Reader 1		
* N* (%)	0 (0%)	712 (100%)
Reader 2		
* N* (%)	5 (1%)	707 (99%)
Reader 3		
* N* (%)	27 (4%)	685 (96%)
Majority Read		
* N* (%) Median Bladder *SUV*_*mean*_^a^ Median Bladder *SUV*_*max*_^a^	2 (0.3%) 26.7 38.6	710 (100%) 12.5 17.0

^a^Using SUV data from Reader 1’s analysis

## Discussion

Here, we conducted both a multi-reader qualitative analysis and a single reader quantitative analysis to determine the potential impact of any urinary activity of ^18^F-flotufolastat in the bladder and ureters on the assessment of ^18^F-flotufolastat-PET images from patients with newly diagnosed and recurrent prostate cancer.

Quantitative findings show the overall urinary activity of ^18^F-flotufolastat to be relatively low, and, while this study was not designed as a head-to-head comparison, the median bladder *SUV*_*max*_ (17.1) and *SUV*_*mean*_ (12.5) recorded here compare favorably with values reported in the literature for other renally excreted PSMA-targeted radiopharmaceuticals (Supplemental Table 2).

Physiological bladder activity is a common feature of PET radiopharmaceuticals, and since activity in the bladder could potentially obscure lesions adjacent to the bladder, various approaches have been used in order to minimize the impact on image interpretation. These include administering intravenous fluids to dilute accumulating activity in the bladder [5], imaging the pelvic region at an early timepoint before bladder accumulation becomes substantial [5, 8], and employing later imaging timepoints after forced diuresis [10, 11]. If clinically necessary, co-administration of a diuretic is suggested in the ^68^Ga-PSMA-11 prescribing information as a method to potentially decrease artifact from ^68^Ga-PSMA-11 accumulation in the urinary bladder and ureters [15]. However, administration of a diuretic may cause discomfort to a patient because of the increased urge to void, which could be of particular significance in prostate cancer patients who may have micturition difficulties due to enlarged intact prostates, or who may suffer from incontinence following surgery. In the LIGHTHOUSE and SPOTLIGHT studies, no patients were co-administered diuretics for scanning purposes.

**Fig. 5:**
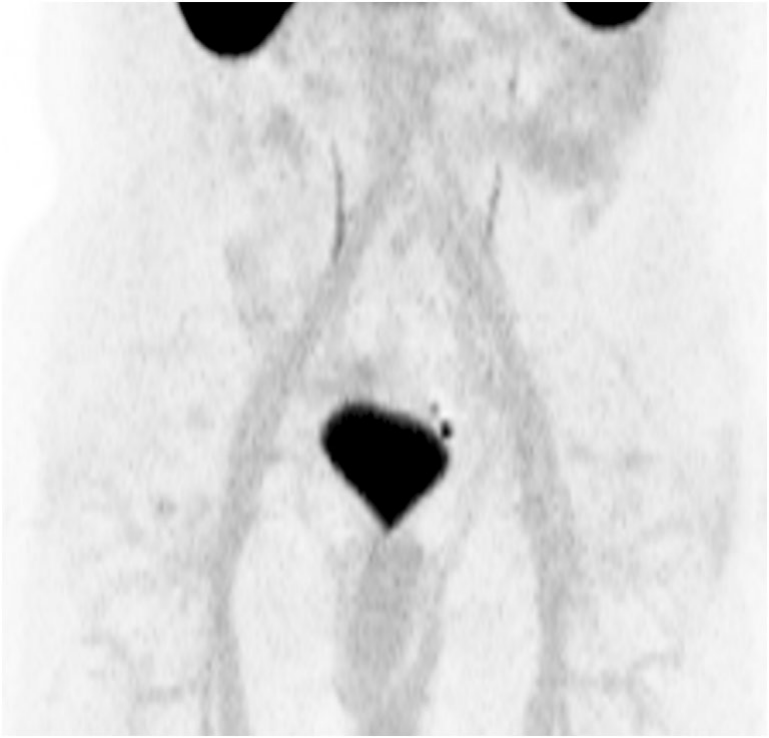
Patient with visible ureters and two pelvic lymph nodes. The figure presents the MIP image (SUV scale 0–10) of a patient with two visible ureters and two separately identifiable left pelvic lymph node metastases

Qualitative findings further demonstrate the minimal impact of ^18^F-flotufolastat urinary activity in both the bladder and ureters on ^18^F-flotufolastat-PET-only image interpretation. By majority read, 96% of scans were considered to be unaffected without consulting other modalities than PET by urinary activity in the bladder (score 0 or 1). However, we note that PET-only assessment of the scans could be affected by urinary activity in up to 13% of the cases, dependent on the reader (3% by majority read). Moreover, in those cases where urinary activity did influence the PET assessment, the median bladder *SUV*_*max*_ and *SUV*_*mean*_ were markedly higher. However, the data acquired did not specify if it was urinary activity in the bladder, in the ureters, or the urethra that interfered with the assessment of the scan. Therefore, the higher median SUVs observed in the bladder do not directly infer that the higher bladder activity obscured a lesion in close proximity to the bladder; rather, it can be taken to be representative of higher urinary tract activity in general. Ureteric activity was absent in more than half of the patients and, as shown in Fig. 2, even when present, had little impact on disease assessment. As shown in Fig. 5, the ureters depicted here do not interfere with identifying the adjacent LN metastases. One of the factors that might interfere with assessment of the scan is a transient focus of urine in the ureter, which could be mistaken for a LN metastasis. In this particular analysis, that focus might not be classified as “stasis of urine” and therefore unclassified as a ureter. However, readers were instructed to assign a qualitative score 2 if they were not able to distinguish between urinary activity and disease. Those cases are therefore captured in the qualitative analysis and, as stated above, are part of the small group of patients (24/712, 3.4%) in which urinary activity interfered with scan assessment. We therefore conclude that the ureteric activity reported here did not interfere with PET-only assessment in the vast majority of cases.

Halo artifacts are a scatter correction artifact which derive from high organ-to-background activity ratios such as between the bladder and surrounding soft tissue, and a positive correlation between organ-to-background ratio and halo size has been reported [6]. Halo artifacts have been reported to occur with other renally cleared radiopharmaceuticals, particularly with PET/MRI [6, 16–18]. Use of diuresis has been reported to assist in reducing this phenomenon [16]. Although not strictly radiopharmaceutical-dependent, their occurrence is due to the physiologic biodistribution of the radiopharmaceuticals, and those with higher bladder accumulation are more likely to be affected [6]. Here, we show that the mean bladder SUVs of ^18^F-flotufolastat are lower than those reported for both ^18^F-DCFPyL and ^68^Ga-PSMA-11, but also that halo artifacts were extremely rare, occurring in fewer than 1% of patients (majority read) undergoing ^18^F-flotufolastat-PET/CT.

There are some limitations to the present analysis. First, the aim of the study was not to identify any lesions that were obscured by the bladder through comparison of urinary activity in the urinary tract to activity in potential lesions, rather to qualitatively assess if urinary activity might interfere with reading. Thus, SUVs in the urinary tract were not compared with those of metastatic lesions, and this should be the focus of future studies. Second, while reader agreement was broadly high across all measured variables, there was some level of disagreement on the presence of halo artifacts (ranged from 0 to 27 across readers). While one reader had a stricter definition of a halo, *i.e.*, a reconstruction artifact deriving from the high activity in the bladder (and finding none), another reader actively searched for more subtle photopenic rim around the bladder that could potentially be called a halo artifact (identifying 27 cases). However, even with the latter, most sensitive definition used, only in 4% of cases a potential halo could be found. Furthermore, the two patients who had a halo artifact as per majority read had a qualitative score of 1 (by all three readers) indicating that the halo artifact did not interfere with image interpretation. While reader agreement was not formally tested in the present analysis, inter-reader comparison data have previously been reported for the blinded reads in the prostate/bed, pelvic LN, and extra-pelvic regions in the LIGHTHOUSE and SPOTLIGHT studies, which show a greater than ≥ 95% and ≥ 75% patient-level inter-reader agreement, respectively [19, 20]. Third, this post hoc analysis was not designed as a head-to-head comparison with other PSMA-PET radiopharmaceuticals, and thus, any comparisons of the median SUV reported here with data from other radiopharmaceuticals reported in the literature should be made with caution. Finally, as stated above, this study did not categorize the level of the urinary tract at which urinary activity interfered with assessment in patients who scored 2 on the qualitative scale. Therefore, it remains unknown to which extent bladder activity interfered with assessing local recurrence, and ureteric activity interfered with assessing pelvic and retroperitoneal LN.

Correctly staging patients is essential to guide patients to the most effective treatment options, and the addition of further accurate tools to the armamentarium of diagnostic options is vital in order to provide for patients across the prostate cancer spectrum. This large dataset from two phase 3 prospective trials suggests ^18^F-flotufolastat-PET urinary activity is relatively low and rarely impacts disease assessment, building on early clinical data [13] that show ^18^F-flotufolastat-PET to have lower average urinary excretion than reported for other renally cleared PSMA-PET radiopharmaceuticals to indicate a potential benefit of this novel radiopharmaceutical.

## Conclusions

Data from this post hoc analysis of ^18^F-flotufolastat scans from two prospective phase 3 trials show that the urinary activity of ^18^F-flotufolastat is relatively low and does not influence disease assessment for the vast majority of patients. Moreover, while this study was not designed as a head-to-head comparison, the median bladder SUVs are lower than values reported in the literature for other renally cleared PSMA-PET radiopharmaceuticals.

### Supplementary Information

Below is the link to the electronic supplementary material.

Supplementary file1 (DOCX 348 KB)

## Data Availability

The data are available from the corresponding author on reasonable request.
