# Supplementary material for: Quantitative and Qualitative Assessment of Urinary Activity of 18F-Flotufolastat-PET/CT in Patients with Prostate Cancer: a Post Hoc Analysis of the LIGHTHOUSE and SPOTLIGHT Studies
Source: Mol Imaging Biol. 2023 Nov 6;26(1):53–60. doi: 10.1007/s11307-023-01867-w (PMC10827967; doi:10.1007/s11307-023-01867-w)
Supplement: Supplementary file 1 — Supplementary file1 (DOCX 348 KB) [file 11307_2023_1867_MOESM1_ESM.docx]

**Supplemental Appendix**

**Supplemental Figure 1.** Bladder activity assessment method


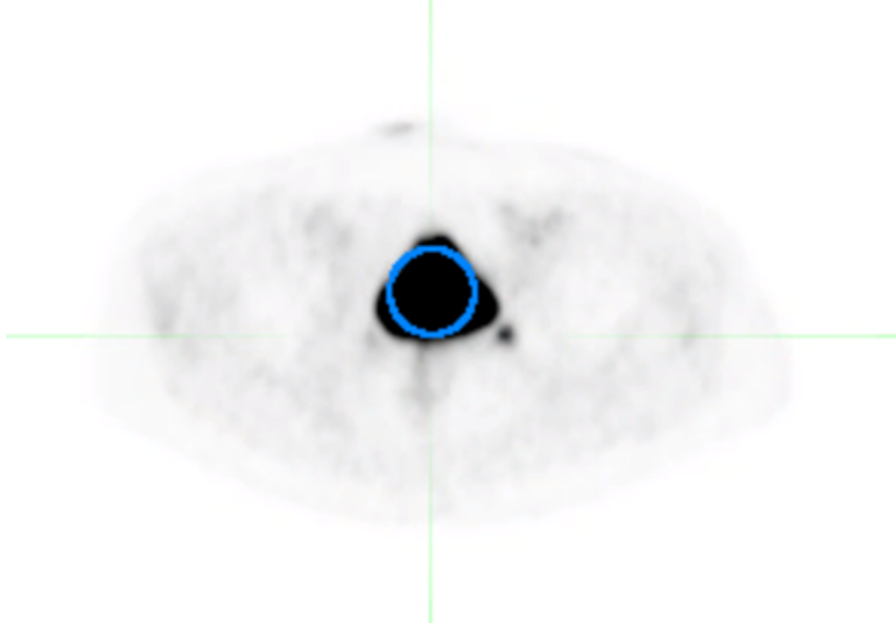


Bladder activity was quantitatively assessed by placing an ROI in the maximum radioactive diameter in the transverse plane in such a way as the ROI diameter was maximized, whilst still being contained within the radioactive extent of the bladder. SUV_max_ and SUV_mean_ from this ROI were recorded. SUV scale 0–10.

**Supplemental Table 1.** Read methodology for the qualitative assessment

| **General** | Readers will re-evaluate all ^18^F-flotufolastat-PET scans acquired in the studies <<LIGHTHOUSE>> and <<SPOTLIGHT>> and will perform qualitative characterization of the urinary excretion of ^18^F-flotufolastat.  Qualitative assessment will be scored based on the extent to which urinary excretion affected assessment of the prostate/ bed and/or for the para-iliac nodes:   - **0** – no or hardly any urinary activity visible; - **1** – urinary activity visible, but distinction between urine and disease possible; - **2** – assessment inhibited by urinary activity.   In order to explore the presence of urinary activity in the ureters the readers will assess the MIP for the presence or absence of linear stasis of activity in keeping with ureteric activity. In order to explore the presence of halo artifacts around the bladder, the readers will assess the transverse slices for the presence or absence of a gross photopenic region around bladder extending significantly beyond the bladder and overlaying the other structures of the pelvis. |
| --- | --- |
| **Directions for use of MIM workstation** | **1.** Open the attenuation corrected PET and CT in the default MIM workflow.  **2.** Adjust the PET SUV window level to 0–10, use this window level for all steps.  **3.** Perform the qualitative score assessment and complete the corresponding section on the results sheet.  a. Review both the MIP and all three planar reconstructions.  b. The CT may be used for anatomical reference only.  **4.** Perform the qualitative assessment of ureteric activity and complete the corresponding section on the results sheet.  a. Review the MIP only.  **5.** Perform the qualitative assessment of halo artifacts and complete the corresponding section on the results sheet.  a. Review the axial PET slices around the bladder only. |
| **Qualitative score read methodology** | **0** – No or hardly any urinary activity visible: light grey activity in the bladder when the scan is being read at the suggested window level of 0-10 (as per reading guideline, unpublished).  **1** – Urinary activity visible, but distinction between urine and disease possible:   - linear activity in the ureters and separately visible nodular-shaped uptake in keeping with LNs or no LNs; - activity in the bladder, but primary or local recurrence or LNs close to bladder clearly   separate from bladder;   - activity in the urethra, easily distinguishable from primary or local recurrence in the prostate bed.   **2** – Assessment inhibited by urinary activity:   - Reader not able to tell (without consulting other modalities) if a single focus of activity is either a lymph node or stasis of urine in the ureter; - Reader unable to separate bladder from disease. |
| **Ureteric activity read methodology** | **Yes** – Linear stasis of urine as visualized on the MIP at the suggested window level of 0–10 (as per reading guideline, unpublished) regardless of the presence or absence of nodular activity (i.e. LNs).  **No** – No linear stasis visible; nodular foci of activity (? urine or LN) would be covered by score 2 (see above). |
| **Halo artifact read methodology** | **Yes** – Gross photopenic region around bladder (as seen on transverse slices) extending significantly beyond the bladder and overlaying the other structures of the pelvis.  **No** – No artifact or only a minor photopenic rim around bladder (as seen on transverse slices) which does not extend beyond the bladder or overly other structures of the pelvis. |
| CT, computed tomography; LN, lymph node; MIP, maximum intensity projection; PET, positron emission tomography; SUV, standardized uptake value. | |

**Supplemental Table 2.** Median SUV_max_ and SUV_mean_ values for ^18^F-flotufolastat from the present study, and as reported in the literature for studies of ^18^F-DCFPyL and ^68^Ga-PSMA-11 without diuretic use.

| Compound | Median SUV_max_ |  | Median SUV_mean_ |
| --- | --- | --- | --- |
| ^18^F-Flotufolastat | 17.1 (n = 712)* |  | 12.5 (n = 712)* |
| ^18^F-DCFPyL | 79.3 (n = 12) [1]  61.7 (n = 51) [2] |  | – |
| ^68^Ga-PSMA-11 | 43.5 (n = 51) [2]  68.4 (n = 50) [3] |  | 25.4 (n = 16) [4]  45.8 (n = 50) [3] |
| *Data from the present analysis | | | |

**References**

1. Giesel FL, Will L, Lawal I, et al. (2018) Intraindividual comparison of ^18^F-PSMA-1007 and ^18^F-DCFPyL PET/CT in the prospective evaluation of patients with newly diagnosed prostate carcinoma: A pilot study. J Nucl Med 59:1076-80.
2. Donswijk ML, Wondergem M, de Wit-van der Veen L, et al. (2022) Effects of furosemide and tracer selection on urinary activity and peri-bladder artefacts in PSMA PET/CT: a single-centre retrospective study. EJNMMI res 12:42.
3. Uprimny C, Bayerschmidt S, Kroiss AS, et al. (2021) Impact of forced diuresis with furosemide and hydration on the halo artefact and intensity of tracer accumulation in the urinary bladder and kidneys on [^68^Ga]Ga-PSMA-11-PET/CT in the evaluation of prostate cancer patients. Eur J Nucl Med Mol Imaging 48:123-33.
4. Kuten J, Fahoum I, Savin Z, et al. (2020) Head- to head comparison of ^68^Ga-PSMA-11 with ^18^F-PSMA-1007 PET/CT in staging prostate cancer using histopathology and immunohistochemical analysis as reference-standard. J Nuc Med 61:527-32.
